# The Selective Phosphoinoside-3-Kinase p110δ Inhibitor IPI-3063 Potently Suppresses B Cell Survival, Proliferation, and Differentiation

**DOI:** 10.3389/fimmu.2017.00747

**Published:** 2017-06-30

**Authors:** Honyin Chiu, Sharmila Mallya, Phuongthao Nguyen, Annie Mai, Leandra V. Jackson, David G. Winkler, Jonathan P. DiNitto, Erin E. Brophy, Karen McGovern, Jeffery L. Kutok, David A. Fruman

**Affiliations:** ^1^Department of Molecular Biology and Biochemistry, University of California, Irvine, Irvine, CA, United States; ^2^Infinity Pharmaceuticals, Inc., Cambridge, MA, United States

**Keywords:** phosphoinoside-3-kinase, lipid kinase, B cell survival, B cell proliferation, B cell differentiation

## Abstract

The class I phosphoinoside-3-kinases (PI3Ks) are important enzymes that relay signals from cell surface receptors to downstream mediators driving cellular functions. Elevated PI3K signaling is found in B cell malignancies and lymphocytes of patients with autoimmune disease. The p110δ catalytic isoform of PI3K is a rational target since it is critical for B lymphocyte development, survival, activation, and differentiation. In addition, activating mutations in *PIK3CD* encoding p110δ cause a human immunodeficiency known as activated PI3K delta syndrome. Currently, idelalisib is the only selective p110δ inhibitor that has been FDA approved to treat certain B cell malignancies. p110δ inhibitors can suppress autoantibody production in mouse models, but limited clinical trials in human autoimmunity have been performed with PI3K inhibitors to date. Thus, there is a need for additional tools to understand the effect of pharmacological inhibition of PI3K isoforms in lymphocytes. In this study, we tested the effects of a potent and selective p110δ inhibitor, IPI-3063, in assays of B cell function. We found that IPI-3063 potently reduced mouse B cell proliferation, survival, and plasmablast differentiation while increasing antibody class switching to IgG1, almost to the same degree as a pan-PI3K inhibitor. Similarly, IPI-3063 potently inhibited human B cell proliferation *in vitro*. The p110γ isoform has partially overlapping roles with p110δ in B cell development, but little is known about its role in B cell function. We found that the p110γ inhibitor AS-252424 had no significant impact on B cell responses. A novel dual p110δ/γ inhibitor, IPI-443, had comparable effects to p110δ inhibition alone. These findings show that p110δ is the dominant isoform mediating B cell responses and establish that IPI-3063 is a highly potent molecule useful for studying p110δ function in immune cells.

## Introduction

Phosphoinoside-3-kinase (PI3K) enzymes are lipid kinases that produce 3′-phosphorylated phosphoinositides, which act as second messengers to relay signals from cell-surface receptors to downstream mediators. The class I PI3Ks produce phosphatidylinositol-3,4,5-triphosphate (PIP_3_) that recruits cytoplasmic proteins to the membrane to drive downstream signaling responses. Class IA PI3Ks are heterodimers composed of two subunits, a regulatory subunit (p85) and one of three catalytic subunits (p110α, p110β, p110δ). The class IB PI3K is composed of unique regulatory subunits (p101 or p84) with the catalytic subunit p110γ. Whereas p110α and p110β are ubiquitously expressed, p110δ and p110γ expression is mainly restricted to leukocytes. The importance of PI3K activation in various cancers has led to development of many small molecule PI3K inhibitors targeting individual isoforms or subgroups (1).

Elevated PI3K signaling is commonly detected in malignant B cells and in peripheral lymphocytes from patients with antibody-driven autoimmune diseases like lupus (2). Both genetic and pharmacological studies have implicated p110δ to be critical for B lymphocyte development, survival, activation, and differentiation (3–5). Moreover, activating mutations in *PIK3CD* encoding p110δ cause a human immunodeficiency known as activated PI3K delta syndrome (APDS), which is associated with chronically activated lymphocytes that undergo apoptosis or senescence (6, 7). Therefore, p110δ has been extensively studied as a potential target for treating B cell malignancies, B cell-mediated autoimmune diseases, and potentially APDS. Impressive responses in clinical trials of idelalisib (previously known as GS-1101 or CAL-101) led to FDA approval of this drug for treatment of certain B cell malignancies (8).

Other p110δ inhibitors have shown activity in animal models of autoimmunity. For example, IC87114 reduced autoantibody production in a rat model of collagen-induced arthritis (9). Another recently developed p110δ inhibitor, AMG319, reduced KLH-specific IgM and IgG production *in vivo* (10) while duvelisib (IPI-145), a dual p110δ/γ inhibitor, showed potent activity in reducing inflammation in collagen-induced arthritis, ovalbumin-induced asthma, and systemic lupus erythematosus rodent models (11). Currently, however, there are no approved treatments targeting p110δ in B-cell-mediated autoimmune diseases. Additional p110δ inhibitors with high potency and selectivity are needed as research tools for B cell biology and as potential lead compounds for B cell-driven diseases. Characterizing the effects of isoform-selective PI3K inhibitors on normal B cell function will provide insight toward finding effective therapeutic windows that can target B cell malignancies while maintaining effective host defense and may justify clinical exploration of these inhibitors in treating B cell-mediated autoimmune disease.

Previous studies have demonstrated that p110δ is not the only PI3K isoform that contributes to B cell function. We used isoform-selective compounds to show that acute inhibition of either p110α or p110β partially reduce signaling and functional responses in activated B cells (12). Genetic analysis has shown partially overlapping roles of p110δ and p110α in B cell development (13). Little is known about the role of the class IB isoform p110γ in B cells. In T cells, p110γ plays a role in early development and is important for trafficking of activated effector cells (14, 15). One study reported that mice lacking both p110δ and p110γ had greater defects in B cell survival and proliferation compared to p110δ knockout alone (16). The effects of chemical p110γ inhibition on B cell function have not been reported.

In this study, we utilized a novel, potent, and selective p110δ inhibitor, IPI-3063 (Table 1) that has good pharmacokinetics in mice (11). Here, we tested the effects of IPI-3063 on mouse B cell survival, proliferation, and differentiation. We found that IPI-3063 is highly potent, modulating B cell responses at low nanomolar concentrations to an extent similar to a pan-PI3K inhibitor. In contrast, a selective chemical inhibitor of p110γ had no effect in various assays of B cell function. We also tested a novel dual p110δ/γ inhibitor, IPI-443 (Table 1), to determine whether p110γ inhibition increases the effects beyond blockade of p110δ alone. Dual inhibition of p110δ/γ with IPI-443 had comparable effects to IPI-3063 on B cell function. These results confirm that p110δ is the dominant isoform that mediates B cell responses to diverse stimuli and establish that IPI-3063 is a highly potent molecule to probe p110δ function in immune cells.

**Table 1:** Summary of IC_50_ values for IPI-3063 and IPI-443 using purified enzymes.

Phosphoinoside-3-kinase isoform	Biochemical IC_50_, nM (*n*)	
	IPI-3063	IPI-443
p110α	1,171 ± 533 (6)	990 ± 695 (6)
p110β	1,508 ± 624 (5)	4,005 ± 2,563 (6)
p110δ	2.5 ± 1.2 (5)	6.3 ± 3.2 (6)
p110γ	2,187 ± 1,529 (4)	23.4 ± 12.3 (6)

## Results

### Inhibition of p110δ, but Not p110γ, Reduces p-AKT in Activated Mouse B Cells

**Figure 1:**
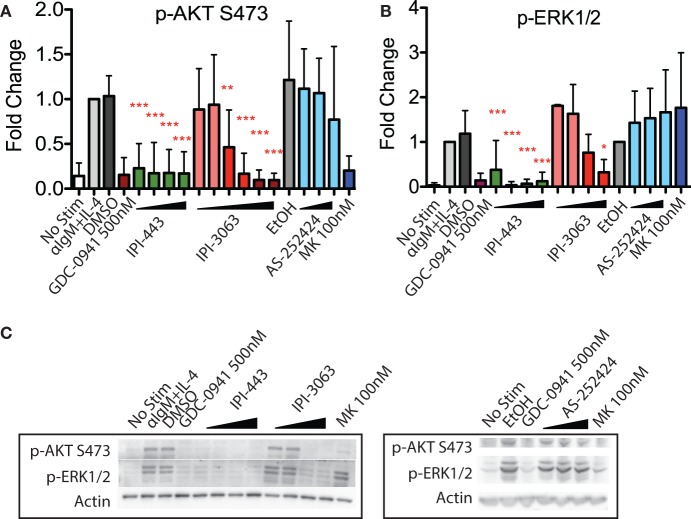
Inhibition of p110δ, but not p110γ, reduces p-AKT and p-ERK1/2 in αIgM + IL-4 stimulated mouse B cells. Purified B cells were pretreated with inhibitors as indicated for 30 min then activated with 5 µg/mL αIgM + 10 ng/mL interleukin-4 (IL-4) for 1 h before harvest for western blot. **(A,B)** Concentrations used were 1 nM, 10 nM, 100 nM, 1 µM for IPI-443 and 0.01, 0.1, 1, 10, 30, 100 nM for IPI-3063. AS252424 concentrations were 100 nM, 300 nM, and 1 µM, and GDC-0941 was at 500 nM. **(B,C)** Concentrations of IPI-3063 used to measure p-ERK1/2 were 0.01, 0.1, 1, and 10 nM. For the graphs in panels **(A,B)**, data were normalized to the stimulated, DMSO 0.1% condition (*P < 0.05, **P < 0.001, ***P < 0.0001 one-way ANOVA with Newman–Keuls multiple comparison test).

IPI-3063 is a p110δ selective compound with an IC_50_ = 0.1 nM in p110δ-specific cell-based assays and cellular IC_50_ values for the other class I PI3K isoforms are at least 1,000-fold higher (Table 2) (11). IPI-443 is a selective p110δ/γ dual inhibitor with a cellular IC_50_ = 0.29 nM for p110δ and IC_50_ = 7.1 nM for p110γ. IPI-443 activity for p110α and p110β is >600-fold less potent compared to activity for p110δ (Table 2). To test the effects of p110γ inhibition, we used AS-252424 compound with a biochemical IC_50_ = 30 nM for p110γ (17). We assessed the effects of both IPI-3063 and IPI-443 on PI3K activity in mouse primary B cells stimulated with αIgM + IL-4, by evaluating phosphorylation of AKT at the serine 473 residue (Figures 1A,C) as well as the phosphorylation of ERK1/2 on Thr202/Tyr204 residues (Figures 1B,C). Cells were treated with a pan-PI3K inhibitor GDC-0941 or with various concentrations of IPI-3063 or IPI-443, or DMSO (0.1%) as the diluent control. Separate cultures were treated with the p110γ inhibitor AS-252424 or its diluent control (0.1% EtOH). MK-2206, an AKT inhibitor, which also reduces AKT S473 phosphorylation, was also used as a control to show ERK1/2 phosphorylation is independent of AKT activity. The p110δ inhibitor, IPI-3063, was very potent in reducing p-AKT (significant effect at 1 nM) while the p110γ inhibitor, AS-252424, had no significant effect on p-AKT signaling. IPI-3063 also reduced p-ERK1/2 with a significant effect at 10 nM, whereas AS-252424 had no significant effect. The dual p110δ/γ inhibitor, IPI-443, was also very potent in decreasing phosphorylation of AKT, with significant effects observed using concentrations as low as 1 nM, which are in the range where p110δ is targeted selectively. These data indicate that both inhibitors are very potent in reducing PI3K signaling output while p110γ inhibition did not have a significant effect. B cells stimulated with LPS showed similar results with p-AKT (Figure S1 in Supplementary Material); however, LPS did not induce ERK1/2 phosphorylation (data not shown). AS-252424 caused dose-dependent inhibition of AKT phosphorylation in bone marrow-derived myeloid cells stimulated with macrophage colony stimulating factor, confirming that this compound inhibits PI3Kγ activity in cells (data not shown).

**Table 2:** Summary of IC_50_ values for IPI-3063 and IPI-443 in isoform-specific cell-based assays.

Phosphoinoside-3-kinase isoform	Cellular IC_50_, nM (*n*)	
	IPI-3063	IPI-443
p110α	1,901 ± 1,318 (4)	901.8 ± 62.4 (3)
p110β	102.8 ± 35.7 (4)	185.2 ± 17.2 (3)
p110δ	0.1 ± 0.01 (6)	0.29 ± 0.03 (4)
p110γ	418.8 ± 117.2 (2)	7.1 ± 0.5 (3)

### IPI-3063 Potently Inhibits B Cell Survival and Proliferation

**Figure 2:**
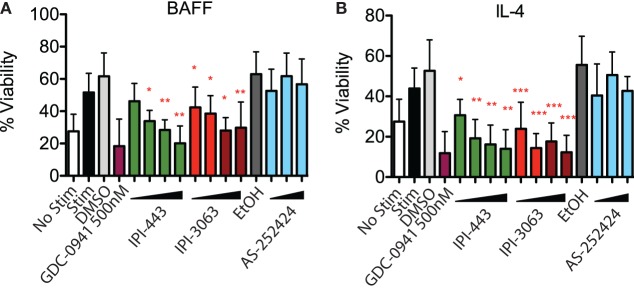
IPI-3063 potently inhibits mouse B cell survival. Total splenocytes were pretreated with inhibitors as indicated, then cultured with 10 ng/mL IL-4 **(A)** or 60 ng/mL of BAFF **(B)** for 48 h. Concentrations used were 1 nM, 10 nM, 100 nM, 1 µM for IPI-443 and 1, 10, 30, 100 nM for IPI-3063. AS252424 concentrations were 100 nM, 300 nM, and 1 µM, and GDC-0941 was at 500 nM. % viability was calculated by measuring % of B220^+^7AAD^−^ cells. Samples were collected by time. (*P < 0.05, **P < 0.001, ***P < 0.0001 one-way ANOVA with Newman–Keuls multiple comparison test).

To assess survival, purified mouse B cells were incubated for 48 h in either B-cell activating factor (BAFF) or interleukin-4 (IL-4) with various concentrations of IPI-3063 and IPI-443. The results showed that both IPI-3063 and IPI-443 reduced BAFF-dependent survival in a dose-dependent manner, approaching the effect of GDC-0941 (Figure 2). The selective PI3K inhibitor IPI-3063 was very potent, achieving a significant decrease in B cell survival when present at 10 nM. IPI-443 significantly decreased survival when added at 1 nM. The p110γ inhibitor AS-252424 had no significant effect on survival. Cells incubated with IL-4 showed similar trends.

**Figure 3:**
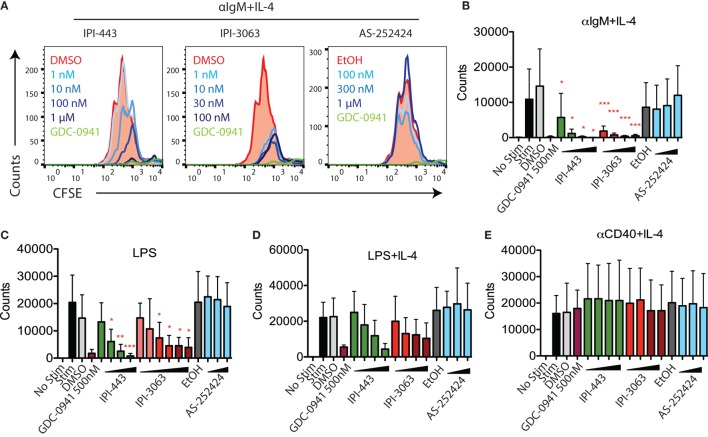
IPI-3063 potently inhibits mouse B cell proliferation. Total splenocytes **(A,B)** or purified B cells **(C–E)** were pretreated with inhibitors for 30 min, then stimulated with **(A,B)** αIgM + IL-4 for 72 h, **(C–E)** LPS + IL-4 for 96 h, LPS for 72 h, or αCD40 + IL-4 for 96 h. Concentrations used were 1 nM, 10 nM, 100 nM, 1 µM for IPI-443 and 1, 10, 30, 100 nM for IPI-3063. AS252424 concentrations were 100 nM, 300 nM, and 1 µM, and GDC-0941 was at 500 nM. The experiments in panel **(C)** include low concentrations of IPI-3063 (0.01 and 0.1 nM) Total numbers were determined by the number of B220^+^7AAD^−^CFSE^lo^ cells. Samples were collected by time (*P < 0.05, **P < 0.001, ***P < 0.0001 one-way ANOVA with Newman–Keuls multiple comparison test).

Next, we evaluated the effects of PI3K inhibitors on B cell proliferation. We stimulated CFSE-stained cells with αIgM + IL-4 or with LPS for 72 h, or with αCD40 + IL-4 or LPS + IL-4 for 96 h. Cells were treated with vehicle or GDC-0941, IPI-3063, IPI-443, or AS-252424 at various concentrations. Histograms of CFSE fluorescence in the cells show that IPI-3063 blocked proliferation in α-IgM + IL-4 stimulated B cells at the lowest concentration tested (1 nM) (Figure 3A). IPI-443 caused a dose-dependent decrease in proliferation, whereas AS-252424 had no effect. We measured the total number of divided cells over multiple experiments and found that IPI-3063 significantly reduced cell accumulation at all concentrations tested (Figure 3B). IPI-443 had a dose-dependent effect starting at 1 nM, with similar effects in LPS-stimulated B cells (Figures 3B,C). In B cells stimulated with LPS + IL-4, the inhibitors had similar trends, but with greater variability (Figure 3D). The inhibitors did not affect B cell proliferation following stimulation with α-CD40 + IL-4 (Figure 3E), consistent with previous data showing PI3K-independent proliferation under these conditions (3). We also measured percent of divided cells in these conditions (Figure S2 in Supplementary Material). α-IgM + IL-4 was the only stimulus where p110δ inhibition significantly reduced the percentage of cells dividing. This analysis also showed that combined inhibition of p110δ/γ by IPI-443 at 1 µM reduced the percentage of divided cells more than inhibition of p110δ only. Overall, these experiments establish that the selective p110δ inhibitor IPI-3063 is a very potent inhibitor of B cell survival and proliferation *in vitro*. In addition, selective p110γ inhibition alone had no effect on B cell survival or proliferation.

**Figure 4:**
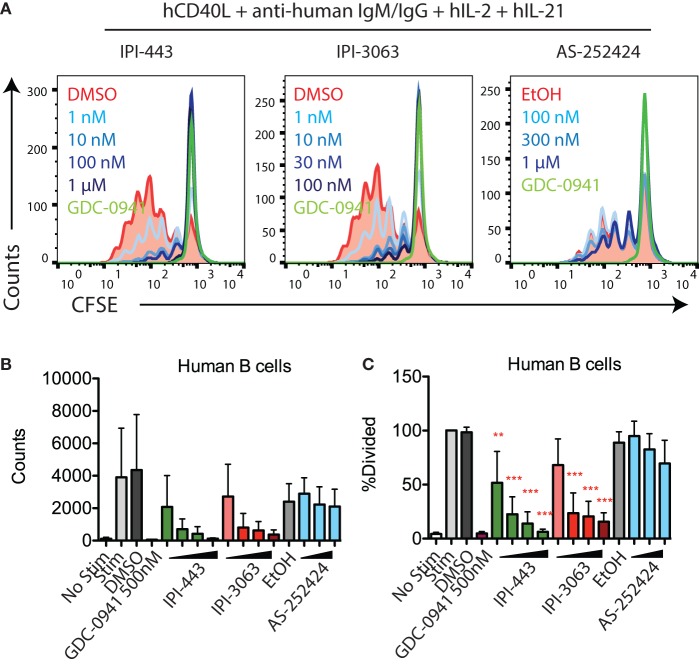
IPI-3063 potently inhibits human B cell proliferation. **(A–C)** Purified human B cells were pretreated with inhibitors for 30 min, then stimulated with human CD40L + anti-human IgM/IgG + human IL-2 + human IL-21 for 120 h. Concentrations used were 1 nM, 10 nM, 100 nM, and 1 µM for IPI-443, and 0.1, 1, 10, and 100 nM for IPI-3063. AS252424 concentrations were 100 nM, 300 nM, and 1 µM, and GDC-0941 was at 500 nM. Total numbers and percent divided were determined by the number of CD19^+^7AAD^−^CFSE^lo^ cells. Samples were collected by time (*P < 0.05, **P < 0.001, ***P < 0.0001 one-way ANOVA with Newman–Keuls multiple comparison test).

We also tested the effects of these inhibitors on human B cell proliferation stimulated with human CD40L + anti-human IgM/IgG + hIL-2 + hIL-21. We stimulated CFSE-stained human B cells for 120 h in the presence of GDC-0941, IPI-3063, IPI-443, or AS-252424 at various concentrations. Histograms of CFSE fluorescence in the cells show that IPI-3063 blocked proliferation at 1 nM (Figure 4A). IPI-443 caused a dose-dependent decrease in proliferation, whereas AS-252424 had no effect. We measured the total number of divided cells and the percent divided over multiple experiments and found that IPI-3063 significantly reduced proliferation starting at 1 nM (Figures 4B,C). IPI-443 also had a dose-dependent effect starting at 1 nM (Figures 4B,C). These experiments extend our findings by showing that, similar to mouse B cells, human B cells treated with the selective p110δ inhibitor IPI-3063 have markedly reduced ability to proliferate *in vitro*. In addition, selective p110γ inhibition also did not impair human B cell proliferation.

### IPI-3063 Potently Promotes Mouse B Cell Antibody Switching and Inhibits Plasmablast Differentiation

**Figure 5:**
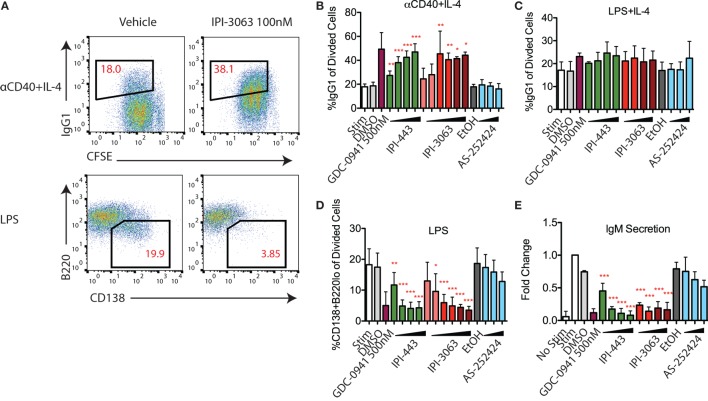
IPI-3063 potently promotes mouse B cell antibody switching and inhibits plasmablast differentiation. Purified B cells were pretreated with inhibitors, then stimulated with αCD40 + IL-4 **(B)** or LPS + IL-4 **(C)** for 96 h to induce switching to IgG1 or LPS **(D)** for 72 h to induce plasmablast differentiation. Class switching to IgG1 was measured by the 7AAD^−^CFSE^lo^B220^+^IgG1^+^ cells [**(A)**, upper panels]. Plasmablast percentages were calculated by % 7AAD^−^CFSE^lo^CD138^+^B220^lo^ population [**(A)**, lower]. Supernatant was harvested **(E)** for IgM ELISA. Concentrations used were 1 nM, 10 nM, 100 nM, 1 µM for IPI-443 and 1, 10, 30, 100 nM for IPI-3063. Low concentrations of IPI-3063 were 0.01 and 0.1 nM **(D)**. AS252424 concentrations were 100 nM, 300 nM, and 1 µM, and GDC-0941 was at 500 nM. Samples were collected by time (*P < 0.05, **P < 0.001, ***P < 0.0001 one-way ANOVA with Newman–Keuls multiple comparison test).

Studies with p110δ inhibitors have shown that low PI3K signaling in B cells promotes antibody class switch recombination (CSR) and decreases differentiation into plasmablasts that secrete low-affinity IgM (18, 19). Activation of mouse primary B cells with αCD40 + IL-4 or LPS + IL-4 both induce IgG1 class switching while treatment with LPS alone induces the plasmablast differentiation fate (Figure 5A). In B cells stimulated with αCD40 + IL-4, IPI-3063 increased the percentage of B220^+^ cells switching to IgG1 starting at 1 nM and approached the effect of GDC-0941 (Figure 5B). IPI-443 followed a similar trend, significantly increasing IgG1 switching at concentrations of 1 nM or higher, while p110γ inhibitor AS-252424 had no effect (Figure 5B). In cells activated with LPS + IL-4, none of the inhibitors significantly increased %IgG1^+^ (Figure 5C).

We also evaluated plasmablast differentiation and IgM secretion in LPS-stimulated cells (Figure 5D). IPI-3063 potently decreased plasmablast differentiation starting at 1 nM, as measured by the percentage of the CD138^+^B220^lo^ population. IPI-443 also caused a dose-dependent decrease starting at 1 nM. In cells treated with IPI-3063 or IPI-443, the highest concentrations inhibited plasmablast differentiation to the same degree as GDC-0941. Measuring IgM secretion by ELISA showed similar trends (Figure 5E). In the plasmablast and IgM secretion assays, the p110γ inhibitor AS-252424 caused no significant effect.

Overall, these results show that IPI-3063 is very potent at increasing B cell antibody class switching to IgG1 in B cells stimulated with αCD40 + IL-4, while potently reducing plasmablast differentiation in cells stimulated with LPS. Combined inhibition of p110δ/γ by IPI-443 had a similar effect that was not significantly greater than inhibition of p110δ only, and importantly, p110γ-specific inhibition did not show any significant effect.

## Discussion

In this study, we have used novel inhibitors of p110δ, p110δ/γ, and p110γ to study how acute inhibition of these isoforms impacts B cell function. Consistent with previous studies (5, 18, 20), our results show that p110δ inhibition decreased several mouse B cell functions including survival, αIgM + IL-4-induced proliferation, and plasmablast differentiation while increasing class switching to IgG1. We also show that these inhibitors are potent at reducing human B cell proliferation *in vitro*. Importantly, additional p110γ inhibition had little to no effect, indicating that the class IB isoform p110γ has a minor role in the function of mature B cells. Both IPI-3063 and IPI-443 were highly potent at inhibiting these B cell functions, with activity in the nanomolar ranges that achieved similar results to the pan-class I inhibitor GDC-0941.

In contrast with a previous study using genetic deletion of the mouse genes encoding both p110δ and p110γ (16), dual inhibition with IPI-443 (achieved at concentrations in the 10–1,000 nM range) did not reduce proliferation and survival more than p110δ inhibition alone in B cells. However, Beer-Hammer et al. also showed that B cell development was impaired at the pre–pro B cell stage when both p110δ/γ are deleted (16). The differences they observed in the double deleted cells compared to single p110δ deletion may be due to developmental defects. In addition, the previous study measured LPS-driven B cell proliferation in the context of total splenocytes, where myeloid cell cytokine production in response to LPS might be a confounding factor. While we did not see any effects with p110γ inhibition or additional effects with dual p110δ/γ inhibition in our assays, these experiments only tested purified B cells *in vitro* and did not test the role of p110γ *in vivo*. Although B cell function is mainly p110δ dependent, p110γ does play an important role in neutrophil, macrophage, and eosinophil recruitment (21) as well as in T cell proliferation and cytokine production (22) and T cell migration (15, 23, 24). Thus, pharmacological inhibition of p110γ *in vivo* could indirectly affect B cell function.

Small molecule inhibitors that are selective for single PI3K isoforms or pairs of isoforms have been highly useful in delineating the shared and distinct functions of PI3K enzymes in diverse cell types (2, 25). Our results show that the selective p110δ inhibitor IPI-3063 and the p110δ/γ dual inhibitor IPI-443 are highly potent, having effects on B cells at the nanomolar range *in vitro*. These data demonstrate that these inhibitors will be useful tools in studying the function of p110δ/γ or p110δ alone. Additional studies will be required to determine whether these or similar compounds will be suitable in treating patients with diseases driven by B cells or other immune cell types in which p110δ and p110γ have key roles.

## Materials and Methods

### PI3K Enzymatic Assay

Human recombinant PI3K-α (cat. no. 14-602-K), -β (cat. no. 14-603-K), -δ (cat. no. 14-604-K), and -γ (cat. no. 14-558-K) were purchased from Millipore. Phosphatidylinositol 4,5 bis phosphate (diC8-PtdIns(4,5)P2) was purchased from Avanti Polar Lipids, Inc. PI3K-α, β, and δ are heterodimers consisting of full length p110α, p110β, or p110δ catalytic subunit and the p85α regulatory subunit. PI3K-γ is a monomer of the p110γ catalytic subunit. Samples of kinase (10 nM—α, β, and δ; 20 nM—γ) were incubated with inhibitor for 30 min at room temperature in reaction buffer (15 mM HEPES pH 7.4, 20 mM NaCl, 1 mM EGTA, 0.02% Tween 20, 10 mM MgCl_2_, 0.2 mg/mL bovine-γ-globulins) followed by addition of ATP/diC8-PtdIns(4,5)P2 mixture to give final concentrations of 3 mM ATP and 500 µM diC8-PtdIns(4,5)P2. Reactions were incubated at room temperature for 2 h, with PI3K activity assessed *via* the Promega ADP-Glo Max assay kit (cat. no. V7002) according to the manufacturer’s instruction. Plates were read on Envision plate reader in luminescence mode.

### pAKT S473 ELISA Assay

Phospho-Akt1 (S473) sandwich ELISA antibody kit (Cell Signaling Technology, cat. no. 7143) was utilized to analyze pAKT signal in cells as described previously by Winkler et al. (11) (for data see Table 2). Briefly, SKOV3 and 786.0 cells were seeded into 96-well cell culture-graded plates at a density of two million per 200 µl culture media per well. Raji and Raw264.7 were seeded at the same density in FBS-free media. After overnight incubation at 5% CO_2_ and 37°C, the cells were treated with inhibitor for 30 min. Raji cells were stimulated with 10 µg/mL anti-human IgM (Jackson ImmunoResearch) for 30 min and Raw264.7 cells with 25 nM C5a (RnD Systems) for 3 min in the presence of inhibitor. SKOV3 and 786.0 cells were not stimulated. Medium was then aspirated and 50 µL/well of ice-cold lysis buffer was added. pAKT level was determined according to the manufacturer’s instruction.

### Mice and Reagents

C57BL/6 mice were bred at the University of California, Irvine, and used at between 6 and 12 weeks of age. All animals were studied in compliance with protocols approved by the Institutional Animal Care and Use Committee of the University of California, Irvine. The p110δ-selective PI3K inhibitor IPI-3063 and p110δ/γ PI3K inhibitor IPI-443 were synthesized at Infinity Pharmaceuticals. These compounds and the pan-PI3K class I inhibitor GDC-0941 (LC laboratories) were dissolved in DMSO. The p110γ PI3K inhibitor AS-252424 (Chemdea) was dissolved in ethanol. Inhibitors were included throughout the indicated cell treatment periods.

### Mouse B Cell Culture

Mouse splenic B cells were purified by negative selection (eBioscience Magnisort Mouse B cell enrichment kit). B-cell purity was >95% as measured by FACS analysis (FACSCalibur and CellQuest software; BD Biosciences) using anti-B220 antibody (BioLegend). Purified B cells were seeded at a final concentration of 0.5 or 0.25 × 10^6^ cells/mL. For plasmablast differentiation, B cells were stimulated with 5 µg/mL LPS (Sigma) for 72 h, and for IgG1 CSR, B cells were stimulated 5 µg/mL anti-CD40 (HM40-3) agonistic antibody (eBioscience), or 5 µg/mL LPS (Sigma), together with 5 ng/mL mIL-4 (R&D Systems) for 96 h. All B cells were cultured in RPMI 1640 supplemented with 10% (vol/vol) heat-inactivated FCS, 5 mM Hepes, 2 mM l-glutamine, 100 U/mL penicillin, 100 µg/mL streptomycin, 50 µM 2-mercaptoethanol.

### Western Blotting Analysis

Analysis was performed on western blots using ImageJ to measure mean fluorescence intensities of each band. Phospho-AKT S473 and phospho-ERK1/2 signal was normalized with actin measurements and fold change was calculated using the stimulated/no drug control.

### Flow Cytometry, CFSE Labeling, and Antibodies

Before cell surface staining, cells were incubated with TruStain fcX in FACS buffer (0.5% BSA + 0.02% NaN3 in 1× HBSS) to block Fc receptors for 10 min on ice. Staining with antibodies was subsequently performed, also with FACS buffer and on ice for 20 min. Flow cytometry antibodies and other reagents used were as follows: B220 (RA3-6B2), IgG1 (A85-1), CD138 (281-2), and 7-Aminoactinomycin D. CFSE labeling of B cells to track proliferation was performed by resuspending cells to a concentration of 10 × 10^6^ cells/mL with a concentration of 2.5 µM CFSE. Flow cytometric data were analyzed using FlowJo software (TreeStar).

### Immunoglobulin ELISA

For cell culture ELISA to measure total IgM, supernatants from purified B cells stimulated with LPS were collected after 3 days and diluted 1:1,000 in 2% (wt/vol) BSA in PBS. Nunc Maxisorp plates (Thermo Fisher) were coated with anti-mouse IgM (RMM-1; BioLegend) at 10 µg/mL in 50 µL of total sample in PBS and allowed to incubate overnight at 4°C. Diluted supernatant samples were incubated on coated plates for 1 h at 37°C. HRP-conjugated rabbit anti-mouse IgM secondary antibody (Zymed) was used.

### Human B Cell Culture

Peripheral blood from normal volunteers was obtained through an Institutional Review Board-approved protocol. Peripheral blood mononuclear cells (PBMCs) were first purified from blood by density gradient centrifugation using Ficoll-Paque. Human B cells were then purified from PBMCs by negative selection (eBioscience Magnisort Human B cell enrichment kit). B-cell purity was increased from 4% to >70% as measured by FACS analysis (FACSCalibur and CellQuest software; BD Biosciences) using anti-CD19 PE conjugated antibody (eBioscience). Purified B cells were seeded at a final concentration of 0.1 × 10^6^ cells/mL and cultured with 2 µg/mL human CD40L (eBioscience) + 5 µg/mL anti-human IgM/IgG (eBioscience) + 100 µg/mL hIL-2 (R&D Systems) + 100 µg/mL hIL-21 (R&D Systems). All B cells were cultured in RPMI 1640 supplemented with 10% (vol/vol) heat-inactivated FCS, 5 mM Hepes, 2 mM l-glutamine, 100 U/mL penicillin, 100 µg/mL streptomycin, 50 µM 2-mercaptoethanol.

## Ethics Statement

Live animals (mice) were studied in compliance with protocols approved by the Institutional Animal Care and Use Committee of the University of California, Irvine.

## Author Contributions

DF designed research and wrote the manuscript. HC designed and performed research and wrote the manuscript. SM, PN, AM, LJ, JD, and EB designed and performed research. DW, KM, and JK designed research and wrote the manuscript.

## Conflict of Interest Statement

DW, JD, EB, KM, and JK were employees and shareholders at Infinity Pharmaceuticals, Inc., at the time of these studies. DF previously served as a consultant for Infinity Pharmaceuticals. The other authors declare that the research was conducted in the absence of any commercial or financial relationships that could be construed as a potential conflict of interest.

## Supplementary Material

Figure S1 Inhibition of p110δ, but not p110γ, reduces p-AKT in LPS-stimulated mouse B cells. Purified B cells were pretreated with inhibitors as indicated for 30 min, then activated with 5 µg/mL LPS for 1 h before harvest for western blot. **(A,B)** Concentrations used were 1 nM, 10 nM, 100 nM, 1 µM for IPI-443 and 1, 10, 30, 100 nM for IPI-3063. AS252424 concentrations were 100 nM, 300 nM, and 1 µM, and GDC-0941 was at 500 nM. **(C)** Concentrations of IPI-3063 were 0.01, 0.1, 1, and 10 nM. For the graph in panel **(A)**, data were normalized to the stimulated, DMSO 0.1% condition (**P* < 0.05, ***P* < 0.001, ****P* < 0.0001 one-way ANOVA with Newman–Keuls multiple comparison test).

Figure S2 IPI-3063 potently inhibits mouse B cell proliferation. Total splenocytes **(A,B)** or purified B cells **(C–E)** were pretreated with inhibitors for 30 min, then stimulated with **(A,B)** αIgM + IL-4 for 72 h **(C–E)** LPS + IL-4 for 96 h, LPS for 72 h or αCD40 + IL-4 for 96 h. Concentrations used were 1 nM, 10 nM, 100 nM, 1 µM for IPI-443 and 1, 10, 30, 100 nM for IPI-3063. AS252424 concentrations were 100 nM, 300 nM, and 1 µM, and GDC-0941 was at 500 nM. The experiments in panel **(C)** include low concentrations of IPI-3063 (0.01 and 0.1 nM). Panel **(A)** shows proliferation of cells by CFSE histograms. Right peak in GDC and IPI-443 1 µM-treated cells represents dead cells. Percent divided were determined by the percent of B220^+^7AAD^−^CFSE^lo^ cells and normalized to no drug treated. Samples were collected by time (**P* < 0.05, ***P* < 0.001, ****P* < 0.0001 one-way ANOVA with Newman–Keuls multiple comparison test).
